# Association of Frailty with recovery from disability among community-dwelling Chinese older adults: China health and retirement longitudinal study

**DOI:** 10.1186/s12877-020-01519-6

**Published:** 2020-03-30

**Authors:** Weihao Xu, Ya-Xi Li, Yixin Hu, Chenkai Wu

**Affiliations:** 1 Geriatric Cardiology Department of the Second Medical Center & National Clinical Research Center for Geriatric Diseases, Chinese PLA General Hospital, Beijing, China; 2 Global Health Research Center, Duke Kunshan University, Academic Building 3038, No. 8 Duke Avenue, Kunshan, 215316 Jiangsu China; 3 Geriatric Health Care Department of The Second Medical Center & National Clinical Research Center for Geriatric Diseases, Chinese PLA General Hospital, Beijing, China; 4 Duke Global Health Institute, Duke University, Durham, NC USA

**Keywords:** Frailty, Recovery, Activities of daily living, Instrumental activities of daily living, Disability, Cohort study

## Abstract

**Backgrounds:**

Little is known about the role of frailty in the recovery process of disability among older adults. We examined the association between frailty and recovery from activities of daily living (ADL) and instrumental ADL (IADL) disability among community-dwelling Chinese older adults.

**Methods:**

Data were from the China Health and Retirement Longitudinal Study. Three waves were used. Participants ≥60 years, had frailty assessment at baseline, and had incident disability in ADL or IADL in 2013, and had disability assessment in 2015 were included. Recovery from ADL and IADL disability were used as outcome measure. Multivariable logistic regression was used to evaluate the potential association between frailty and recovery from ADL or IADL.

**Results:**

We included 516 and 598 participants in the ADL and IADL analysis, respectively. In total, 237 participants recovered from ADL disability and 293 recovered from IADL disability. Nearly half of the non-frail persons recovered from ADL disability, while less than one-quarter of the frail persons had recovery. Over half of the non-frail persons had IADL disability recovery, while only 30% of the frail recovered. After adjustment, the odds of recovery from ADL disability were 59% (95% confidence interval [CI]: 1, 83%) lower among frail participants than those who were non-frail; the odds of recovery from IADL disability were 52% lower among frail persons than those who were non-frail and the association did not reach statistical significance.

**Conclusions:**

Frailty is an independent predictor of poor recovery from disability among nondisabled community-dwelling older adults in China.

## Background

The prevalence of disability in activities of daily living (ADLs) and instrumental ADL (IADLs) has declined during the past several decades in China [1–3]. This promising trend, however, appears to have slowed recently, primarily due to China’s rapidly growing older population. In 2013, over 202 million people were over the age of 60 years in China and the number is projected to nearly double by 2040 (402 million) [4, 5]. The number of disabled older adults was 37.5 million in 2013, and this number will reach 100 million in 2050 [6]. Disability in ADL and IADL is associated with increased risk of hospitalization, lower quality of life, and shorter life expectancy, placing a substantial burden on older people, their caregivers, and the health care system [7–10].

A growing body of literature has suggested that disability is a dynamic rather than static process and transitions among different states of disability are common [11–15]. Prior studies have identified frailty—a clinical syndrome characterized by decreased resilience to stressors and high vulnerability for adverse health outcomes—as a risk factor for poor recovery of independence in ADL among U.S. older adults [16]. However, to our knowledge, little is known about the role of frailty in the recovery process of disability among older adults in developing countries including China. In addition, while previous studies focused on ADL recovery, much less attention has been paid to IADL recovery. ADL and IADL measure different aspects of functional capacity of older adults. ADL includes basic and core self-care tasks (e.g., eating, dressing, bathing), while IADL represents more complicated and higher-level tasks that are necessary for independently adapting to the living environment (e.g., cooking, shopping, taking medication) [17, 18]. Both theory and empirical evidence suggest a hierarchical relationship between IADL and ADL, with older persons first declining in IADL function [19]. Therefore, identification of factors associated with IADL recovery is critical for designing effective interventions in the early stage of the recovery process among the disabled persons.

In the present study, we examined the association between frailty and recovery from ADL and IADL disability among community-dwelling Chinese older adults without pre-existing disability from the China Health and Retirement Longitudinal Study (CHARLS). We hypothesized that frail older adults would have lower ability to recover from disability than non-frail ones.

## Methods

### Data and participants

Data are from the CHARLS, an ongoing longitudinal cohort study of a nationally representative sample of community-dwelling adults from 28 provinces of China. A total of 17,708 residents aged 45 years or older were interviewed at baseline (2011–2012), with a response rate at 80.5%, and followed every two years afterwards (2013–2014 and 2015–2016 waves). All participants gave informed consent; the protocol was approved by the Ethical Review Committee at the Peking University. Further details about the recruitment strategies and study design have been published elsewhere [20].

Frailty was measured at baseline (2011–2012); data on disability were collected at baseline and two follow-ups. We included participants who (i) were ≥ 60 years old, (ii) had frailty assessment at baseline, (iii) had no disability in 2011, (iii) were disabled in 2013, and (iv) had disability assessment in 2015. Among 611 participants who had frailty assessment and were not disabled in ADL in 2011, and developed ADL disability in 2013, we excluded 95 individuals who were lost to follow-up, resulting to an analytic sample of 516 for ADL analysis. Among 701 participants who had frailty assessment and were not disabled in IADL in 2011, and developed IADL disability in 2013, 103 individuals were lost to follow-up, leading an analytic sample of 598 for IADL analysis.

### Frailty

Frailty was assessed by a modified version of the Fried’s Physical Frailty Phenotype (PFP) [21], which was originally developed in the Cardiovascular Health Study. Five criteria were included: slowness, weakness, exhaustion, inactivity, and shrinking (Table 1). This assessment has been developed and validated in the CHARLS to examine the epidemiology and natural history of frailty among community-dwelling older adults in China [22–24].

**Table 1:** Cut-points for defining slowness and weakness

Gait speed over a 2.5-m course:		
	Men with height ≤ 163 cm	≤0.45 m/s
Slowness	Men with height > 163 cm	≤0.48 m/s
	Women with height ≤ 163 cm	≤0.36 m/s
	Women with height > 163 cm	≤0.43 m/s
Grip strength (kg):		
	Men with BMI ≤ 20.6 kg/ **m**^**2**^	≤25.2 kg
	Men with BMI 20.6–23.2 kg/ **m**^**2**^	≤28.5 kg
	Men with BMI 23.2–25.9 kg/ **m**^**2**^	≤30.0 kg
Weakness	Men with BMI ≥ 25.9 kg/ **m**^**2**^	≤30.0 kg
	Women with BMI ≤ 20.0 kg/ **m**^**2**^	≤15.0 kg
	Women with BMI 20.0–22.1 kg/ **m**^**2**^	≤17.5 kg
	Women with BMI 22.1–24.8 kg/ **m**^**2**^	≤17.5 kg
	Women with BMI > 24.8 kg/ **m**^**2**^	≤20.0 kg

Slowness was measured by usual gait speed over a 2.5-m course. Two trials were performed and the average walking time was used to calculate gait speed (m/s). Slowness was defined as sex- and height-specific gait speed ≤20th percentile of the weighted sample distribution [24]. Handgrip strength was measured by dynamometer. Two trials for each hand were performed, and the maximum of four trials was used. Weakness was defined as handgrip strength ≤20th percentile of the weighted sample distribution, adjusting for sex and body mass index (BMI) [24]. Self-reported exhaustion was assessed by two questions in the Center for Epidemiological Studies-Depression scale (CES-D) scale [25]: I felt everything I did was an effort.” and “I could not get going.” Exhaustion was identified if a person answered “A moderate amount of time; 3 to 4 days” or “Most of the time; 5 to 7 days” to either of the two questions. Physical inactivity was defined if a person self-reported that he/she walked < 10 min continuously during a usual week. Shrinking was identified if a person reported losing ≥5 kg in prior year or had a BMI ≤ 18.5 kg/m^2^ (calculated based on measured standing height and body weight).

Frailty was categorized into non-frail, prefrail, and frail according to the number of criteria met. Non-frail was defined when none of the criteria was met; prefrail was defined when one or two criteria were met; frail was defined when three to five criteria were met.

### Outcomes

Disability was assessed using five ADL tasks (dressing, bathing, eating, getting out of bed, and toileting) and five IADL) tasks (preparing hot meals, doing household chores, shopping, managing assets, and taking medications), by asking “Do you have difficulty in” performing the task? Participants who answered “I have difficulty but can still do it”, “Yes, I have difficulty and need help”, or “I cannot do it” to one or more tasks were considered having ADL or IADL disability. Recovery from ADL (or IADL) disability was defined as regaining independence in all ADLs (or IADLs) in 2015 after being disabled in 2013.

### Covariates

Demographic factors included age, sex, education (no formal education, did not finish elementary school, sishu or elementary school, or middle school or higher), and marital status (married vs. not married). Smoking status was categorized as never, former or current smokers. Body mass index (BMI) was calculated as weight (kilograms) divided by height (meters) squared, and classified into underweight (BMI < 18.5), normal (BMI = 18.5–24.0), overweight (BMI = 24.0–28.0), and obese (BMI ≥ 28.0) [26]. History of hypertension, cardiac disease (coronary heart disease, angina, heart failure, myocardial infarction, and other heart diseases), stroke, diabetes, chronic lung diseases, kidney disease, digestive disease, liver disease, cancer (not including minor skin cancers), and arthritis or rheumatism was assessed based on self-reported physician diagnosis. The total number of chronic conditions, ranging from 0 to 10, was used in further analyses. Cognitive function was assessed by the Telephone Interview for Cognitive Status (TICS) [27, 28], a telephone-based cognitive screening instrument. The reliability and validity of the TICS has been demonstrated in different populations and settings [29–32]. Depression was assessed using the modified 10-item CES-D scale [13] excluding two items used for identifying exhaustion (a frailty component) [20]. Severe disability (vs. mild) was defined as having difficulty in ≥2 ADLs or IADLs.

### Statistical analysis

We first described the baseline characteristics of participants included in the ADL analysis (*n* = 516) and IADL analysis (*n* = 598), separately. We used logistic regression models to examine the association between frailty (non-frail, prefrail, and frail) and recovery from disability. Age, sex, and disability severity (severe vs. mild) were included in the minimally adjusted models; education, marital status, BMI, smoking status, depression, cognitive function, and number of chronic conditions were additionally adjusted in the fully adjusted models. ADL and IADL were analyzed separately. In addition, we examined the association between frailty and recovery from IADL disability among 336 persons who had incident IADL disability in 2013 but did not have any difficulty in ADLs. All covariates were measured at the time of frailty assessment instead of at the most recent assessment of covariates prior to the onset of incident disability because covariates measured after frailty could be in the causal pathway between frailty and disability recovery. Adjustment for such mediators could lead to underestimation of the overall effect of frailty.

All tests were two-sided with a significance level of 0.05. We performed all analyses using Stata 15.0 (Stata Corp, College Station, TX).

## Results

### Sample description

We included 516 and 598 participants in the ADL and IADL analysis, respectively. Among participants included in the ADL analysis, 62.0% were 60–69 years of age, 57.4% were female, and 74.2% were married/living together; 37.8, 55.4, and 6.8% were non-frail, prefrail, and frail, respectively (Table 2). The distribution of socio-demographic, lifestyle, and health characteristics was similar among persons included in the IADL analysis.

**Table 2:** Characteristics of study participants

	ADL analysis	IADL analysis
Characteristics	***N*** **= 516**	***N*** **= 598**
Age, years, No (%)		
60–64	202 (39.1)	229 (38.3)
65–69	118 (22.9)	142 (23.7)
70–74	96 (18.6)	117 (19.6)
75–79	68 (13.2)	78 (13.0)
80+	32 (6.2)	32 (5.4)
Female, No. (%)	296 (57.4)	322 (53.8)
Education, No. (%)		
No formal education/illiterate	216 (41.9)	259 (43.3)
Did not finish elementary school	116 (22.5)	132 (22.1)
Sishu or elementary school	135 (26.2)	137 (22.9)
Middle school or above	49 (9.5)	70 (11.7)
Marital Status, No. (%)		
Married/living together	383 (74.2)	469 (78.4)
Smoking status, No. (%)		
Never	314 (60.9)	357 (59.7)
Former	58 (11.2)	72 (12.0)
Current	144 (27.9)	169 (28.3)
Body mass index, kg/ m^2^		
Underweight, No. (%)	56 (10.9)	70 (11.7)
Normal, No. (%)	275 (53.3)	330 (55.2)
Overweight, No. (%)	127 (24.6)	135 (22.6)
Obese, No. (%)	58 (11.2)	63 (10.5)
Number of chronic conditions, mean (SD)	1.6 (1.2)	1.5 (1.2)
Cognitive function ^a^, mean (SD)	10.2 (5.5)	10.4 (5.4)
Frailty, No. (%)		
Non-frail	195 (37.8)	228 (38.1)
Prefrail	286 (55.4)	330 (55.2)
Frail	35 (6.8)	40 (6.7)
Severe disability ^b^, No. (%)	199 (38.6)	280 (46.8)

Abbreviations: *ADL* activities of daily living, *IADL* instrumental activities of daily living; *SD* standard deviation

^a^ Cognitive function was measured by the modified mini-mental status examination

^b^ Severe disability was defined as having difficulty in two or more ADL/IADL

### Association between frailty and recovery from ADL disability

Of the 516 participants who had incident ADL disability, 237 (45.9%) recovered within 2 years (Table 3). Older adults with different levels of frailty had different likelihood of recovering from ADL disability. Nearly half of the non-frail persons recovered, while only about one-quarter of the frail persons had recovery. After multivariable adjustment, the odds of recovery from ADL disability were 59% (95% confidence interval [CI]: 1, 83%) lower among frail participants than those who were non-frail. We did not observe statistically significant difference in recovery between non-frail and prefrail persons.

**Table 3:** Frailty status and recovery from ADL disability

*N* = 516	Recovered No. (%)	Odds ratio (95% CI)	
		Minimally Adjusted ^a^	Fully Adjusted ^b^
Total	237 (45.9)		
Frailty			
Non-frail	97 (49.7)	Ref.	Ref.
Prefrail	131 (45.8)	0.94 (0.65, 1.38)	0.93 (0.62, 1.39)
Frail	9 (25.7)	0.45 (0.19, 1.03)	0.41 (0.17, 0.99)

Abbreviations: *CI* confidence interval, *ADL* activities of daily living

Notes: Participants who died in the following visit after the onset of incident disability were included and considered not to recover. Participants who were alive but not interviewed in the following visit after the onset of incident disability were excluded

^a^ Age, sex, and severity of ADL disability (1 vs. 2+ difficulties)

^b^ Age, sex, severity of ADL disability (1 vs. 2+ difficulties), education, marital status, BMI, smoking status, CESD, cognition, and number of chronic conditions

### Association between frailty and recovery from IADL disability

Recovery from IADL disability within 2 years was observed for 293 (49.0%) of the 598 newly disabled participants (Table 4). The likelihood of recovery decreased steadily from non-frail to frail. Over half of the non-frail persons had recovery, while only 30% of the frail recovered. Among females, 58.1% of the frail, 45.7% of the prefrail, and 24.0% of the non-frail recovered from IADL disability. The association was weaker among males; 50.0, 49.7, and 40% of the frail, prefrail, and non-frail recovered, respectively. After adjusting for age, sex, and severity of IADL disability, the odds of recovery were 58% (95% CI: 10, 80%) lower among frail participants than the non-frail. In the fully adjusted model, the association between frailty and recovery from IADL disability was slightly attenuated. The odds of recovery were 52% lower among frail persons than those who were non-frail and the association did not reach statistical significance.

**Table 4:** Frailty status and recovery from IADL disability

*N* = 598	Recovered No. (%)	Odds ratio (95% CI)	
		Minimally Adjusted ^a^	Fully Adjusted ^b^
Total	293 (49.0)		
Frailty			
Non-frail	124 (54.4)	Ref.	Ref.
Prefrail	157 (47.6)	0.79 (0.56, 1.12)	0.88 (0.61, 1.27)
Frail	12 (30.0)	0.42 (0.20, 0.90)	0.48 (0.21, 1.07)

Abbreviations: *CI* confidence interval, *IADL* instrumental activities of daily living

Notes: Participants who died in the following visit after the onset of incident disability were included and considered not to recover. Participants who were alive but not interviewed in the following visit after the onset of incident disability were excluded

^a^ age, sex, and severity of IADL disability (1 vs. 2+ difficulties)

^b^ age, sex, severity of IADL disability (1 vs. 2+ difficulties), education, marital status, BMI, smoking status, CESD, cognition, and number of chronic conditions

Of the 336 persons who were newly disabled in IADL but were free of ADL disability, 196 (58.3%) recovered within 2 years (Table 5). The chance of recovery differed among persons with different levels of frailty; 67.2, 53.9, and 40.0% of the non-frail, prefrail, and frail participants had recovery, respectively. In the adjusted models, prefrail and frail persons had lower odds of recovery than the non-frail, although the associations were not statistically significant.

**Table 5:** Frailty status and recovery from IADL disability among persons who were not disabled in ADL

*N* = 336	Recovered No. (%)	Odds ratio (95% CI)	
		Minimally Adjusted ^a^	Fully Adjusted ^b^
Frailty			
Non-frail	90 (67.2)	Ref.	Ref.
Prefrail	98 (53.9)	0.60 (0.36, 0.96)	0.66 (0.40, 1.09)
Frail	8 (40.0)	0.45 (0.16, 1.23)	0.52 (0.17, 1.59)

Abbreviations: *CI* confidence interval

Notes: Participants who died in the following visit after the onset of incident disability were included and considered not to recover. Participants who were alive but not interviewed in the following visit after the onset of incident disability were excluded

^a^ Age, sex, and severity of IADL disability (1 vs. 2+ difficulties)

^b^ Age, sex, severity of IADL disability (1 vs. 2+ difficulties), education, marital status, BMI, smoking status, depression, cognition measured by mini-mental status examination, and number of chronic conditions

## Discussion

In this community-based prospective cohort study, we found that frailty was associated with poor recovery from ADL and IADL disability among newly disabled Chinese older adults; once they were newly disabled, non-frail elders had a higher chance of recovering were than the frail. These results provided additional evidence supporting the importance of frailty—a clinical syndrome of reduced reserve to stressors—in the recovery process of disability among older adults.

Our results were in line with several prior studies conducted in the US, showing that frail older adults were less likely to regain independence of ADL after being disabled than the non-frail. Using data from two large U.S. cohort studies—Cardiovascular Health Study and Health and Retirement Study, we demonstrated that frailty was an independent risk factor for poor recovery of ADL function [16]. Using data from 457 moderately or severely disabled community-dwelling older women enrolled in the Women’s Health and Aging Study, Boyd and colleagues showed that frailty was an independent risk factor for decline in ADL independence after a hospitalization [33]. In addition to frailty assessments incorporating multiple components (e.g., physical frailty phenotype approach), several studies have demonstrated the predictive value of individual frailty components, including slow gait speed, low physical activity level, and significant weight loss, for recovery after being disabled among the elderly [13, 14, 34]. The present study extended previous research by highlighting the importance of frailty in the recovery process of ADL disability among Chinese older adults, which constitute the largest older population in the world.

There was suggestive evidence that frailty is associated with poor recovery from IADL disability among newly disabled Chinese older adults. We found that over half of the non-frail persons regained independence in IADL function, whereas only three out of ten frail one recovered. Our study builds on several earlier investigations examining risk factors for IADL recovery. In a study of older adults from five communities in the UK, Seidel et al. found that poor self-reported health and high burden of comorbidity were associated with poor recovery of IADL function [35]. Using data from 1656 persons aged 50 years or above in the UK, d’Orsi and colleagues found that vigorous physical activity and good self-rated memory were associated with higher likelihood of IADL recovery [36]. The present study provides new insight into understanding of the recovery process of IADL disability. The association between frailty and recovery from IADL disability was more evident among older women than men. One plausible explanation is that performing IADL tasks—for example, preparing hot meals and doing household chores—may be challenging even for non-disabled older men because women usually take on these domestic responsibilities. In other words, some older men were classified as being IADL disabled because they did not have the knowledge or skills to perform these activities.

We found that frailty precluded disability recovery—frail older adults were much less likely to recover from disability than the nonfrail. There are several plausible explanations for this phenomenon. First, one defining feature of frailty is reduced reserve and increased vulnerability to stressors. Frail persons with incident disability have compromised ability to deal with this daily stressor and, therefore, less likely to regain independence. Frailty involves declines in gait speed and muscle strength, both of which are critical for performing ADL and IADL. Second, older adults who are frail often have other health problems—such as poor nutrition status, increased level of inflammation—that might hamper disability recovery [37–39]. Therefore, interventions targeting both frailty or frailty components and co-occurring health issues may be important for disabled older adults to regain independence.

Strength of our study include its use of nationally representative sample, prospective design, well validated frailty assessment, comprehensive measurements of confounders, and heterogeneous socio-demographic composition of the study participants. To our knowledge, this study is the first to examine the association between frailty and recovery of ADL and IADL functions among older adults in China. This study is not without limitations. First, the frailty assessment was only administered once; we could not rule out the possibility that there would be unobserved transitions during the follow-up period and we were unable to differentiate transient frailty (more subject to measurement error) and persistent frailty. In addition, we excluded elders who were initially disabled because of the fundamental differences between primary frailty and secondary frailty induced by disability. Moreover, although the modified version of the PFP approach has been validated among Chinese older adults, modifying the criteria could potentially lead to misclassification of frailty status [40]. Furthermore, both ADL and IADL performance were assessed based on self-reports by the participants, which might be different from objective measures. However, it has been demonstrated that the concordance between self-reported disability and objective measures is high [41–43]. Lastly, residual confounding is an inevitable issue in observational studies.

## Conclusion

In summary, we examined the association between frailty, assessed by the PFP scale, and recovery from ADL and IADL disability among community-dwelling older adults in China. Our results provided additional evidence that frailty is an independent predictor of regaining independence after being disabled and demonstrated the construct validity of the PFP scale for assessing frailty, a clinical syndrome characterized by reduced reserve and resilience to stressors. Most of the frailty instruments were validated for predictive validity only. We found that frailty, as assessed by the PFP approach, independently modified older adults’ ability to recovery from disability. This type of empirical investigation has been considered the best way to evaluate the validity of a frailty assessment [44]. Taken together with previous work [45–47], there is strong evidence of the validity of the PFP approach for measuring frailty. Assessment of frailty may help clinicians to better focus their efforts in promoting functional recovery after experiencing a stressful event.

## Data Availability

The raw data is available on website (http://charls.pku.edu.cn/en).

## Abbreviations

ADL: Activities of daily life
IADL: Instrumental activities of daily life
CI: Confidence interval
CHARLS: China Health and Retirement Longitudinal Study
PFP: Physical frailty phenotype
BMI: Body mass index
CES-D: Center for epidemiologic studies - depression Scale
TICS: Telephone interview for cognitive status
